# Expression of survivin detected by immunohistochemistry in the cytoplasm and in the nucleus is associated with prognosis of leiomyosarcoma and synovial sarcoma patients

**DOI:** 10.1186/1471-2407-10-65

**Published:** 2010-02-24

**Authors:** Helge Taubert, Chris Heidenreich, Hans-Jürgen Holzhausen, Antje Schulz, Matthias Bache, Matthias Kappler, Alexander W Eckert, Peter Würl, Ingo Melcher, Kathrin Hauptmann, Steffen Hauptmann, Klaus-Dieter Schaser

**Affiliations:** 1 Department of Oral and Maxillofacial Plastic Surgery, Martin-Luther-University Halle- Wittenberg, Halle, Germany; 2 Institute of Pathology, Martin-Luther-University Halle-Wittenberg, Halle, Germany; 3 Section for Musculoskeletal Tumor Surgery, Center for Musculoskeletal Surgery, Charité - University Medicine, Berlin, Germany; 4 Department of Radiotherapy, Martin-Luther-University Halle-Wittenberg, Halle, Germany; 5 Malteser St. Franziskus Hospital gGmbH, Flensburg, Germany; 6 Department of Pathology, Charité - University Medicine, Berlin, Germany

## Abstract

**Background:**

Survivin, a member of the inhibitor of apoptosis-protein family suppresses apoptosis and regulates cell division. It is strongly overexpressed in the vast majority of cancers. We were interested if survivin detected by immunohistochemistry has prognostic relevance especially for patients of the two soft tissue sarcoma entities leiomyosarcoma and synovial sarcoma.

**Methods:**

Tumors of leiomyosarcoma (n = 24) and synovial sarcoma patients (n = 26) were investigated for their expression of survivin by immunohistochemistry. Survivin expression was assessed in the cytoplasm and the nucleus of tumor cells using an immunoreactive scoring system (IRS).

**Results:**

We detected a survivin expression (IRS > 2) in the cytoplasm of 20 leiomyosarcomas and 22 synovial sarcomas and in the nucleus of 12 leiomyosarcomas and 9 synovial sarcomas, respectively. There was no significant difference between leiomyosarcoma and synovial sarcoma samples in their cytoplasmic or nuclear expression of survivin. Next, all sarcoma patients were separated in four groups according to their survivin expression in the cytoplasm and in the nucleus: group 1: negative (IRS 0 to 2); group 2: weak (IRS 3 to 4); group 3: moderate (IRS 6 to 8); group 4: strong (IRS 9 to 12). In a multivariate Cox's regression hazard analysis survivin expression detected in the cytoplasm or in the nucleus was significantly associated with overall survival of patients in group 3 (RR = 5.7; P = 0.004 and RR = 5.7; P = 0.022, respectively) compared to group 2 (reference). Patients whose tumors showed both a moderate/strong expression of survivin in the cytoplasm and a moderate expression of survivin in the nucleus (in both compartments IRS ≥ 6) possessed a 24.8-fold increased risk of tumor-related death (P = 0.003) compared to patients with a weak expression of survivin both in the cytoplasm and in the nucleus.

**Conclusion:**

Survivin protein expression in the cytoplasma and in the nucleus detected by immunohistochemistry is significantly associated with prognosis of leiomyosarcoma and synovial sarcoma patients.

## Background

Leiomyosarcoma and synovial cell sarcoma are two of the most common malignant soft tissue tumors. Despite survival rates have improved in the past two decades due to advanced treatment with primary radical surgery, along with chemotherapy and radiation, long term prognosis continues to be poor. For instance, synovial sarcoma patients with non-metastatic surgically resected disease are reported to have a 5-year overall survival and the 5-year metastasis-free survival of not more than 71% and 51%, respectively [1]. These survival rates did only tend to result in better outcomes if chemotherapy was performed; clearly underscoring the absolute need for identification of prognostic relevant factors. These factors, possibly assisting in prediction of disease specific prognosis, may help to evaluate the risk for local and systemic recurrence and allow stratifying patients to different treatment strategies.

Among those factors survivin has attracted major interest as it was shown to be strongly overexpressed in a vast majority of cancers, and it is one of the most tumor-specific human gene products [2]. Survivin belongs to two major protein families, the inhibitor of apoptosis and the chromosomal passenger families thereby playing an important role for both regulation of cell death and of cytokinesis [3-7]. Recently, survivin has been considered as putative stem cell marker (reviewed in [8]). A correlation between survivin detection and prognosis of tumor patients has been described for many different cancers (reviewed in [9]). However, there are also reports indicating survivin expression is a favourable prognostic marker (reviewed in [10]). Only a few studies investigated the correlation of survivin protein expression with prognosis in sarcomas as it has been described as prognostic marker for osteosarcomas [11-13]. Nuclear localization of survivin expression was significantly correlated with a prolonged survival but cytoplasmic staining showed no correlation with patients' outcome [11]. In contrast, in another study, survivin expression was significantly associated with the PCNA-labelling index, which was correlated with the histological grades of osteosarcoma [12]. This result rather confirms a role of survivin in inhibiting apoptosis and affecting tumor progression [13]. We investigated survivin expression on the RNA level (qRT-PCR) and on the protein level (ELISA, Western hybridization) in a group of different soft tissue sarcomas including a few leiomyosarcomas and synovial sarcomas, previously. Elevated survivin RNA and protein level were significantly correlated with a poor prognosis of STS patients [9,14]. RNA-Expression of survivin and two other stem cell-associated genes (Hiwi, hTERT) was correlated with a 15.5-fold increased risk of tumor-related death for soft tissue sarcoma patients [15]. There are only two reports that studied survivin protein expression in soft tissue sarcomas by immunohistochemistry but without correlating results with prognosis [16,17]. Caldas et al. could show that over 80% of primary rhabdomyosarcoma tumors expressed survivin and Tabone-Eglinger et al. found survivin protein expressed in all investigated malignant peripheral nerve sheath tumors [16,17]. This study aimed for the first time to analyse expression of survivin protein in the soft tissue entities leiomyosarcoma and synovial sarcoma by immunohistochemistry. In addition, to evaluate the prognostic impact of survivin-expression either detected in the cytoplasm or in the nucleus for leiomyosarcoma and synovial sarcoma patients.

## Methods

### Patients

Twenty-four leiomyosarcoma and 26 synovial sarcoma patients were included in this study. All patients gave written informed consent (Institute of Pathology, University of Halle; Department of Surgery 1, University of Leipzig; Institute of Pathology, Charite - University Medicine, Berlin; Center for Musculoskeletal Surgery, Charité - University Medicine, Berlin). The study was approved by the Local Ethics Committee from the Charite Berlin (EA2/079/07) and the Ethics Committee from the Medizinische Fakultät MLU Halle. The research carried out on humans is in compliance with the Helsinki Declaration. Our study group included 21 males and 29 female patients. The tumors were classified according to the van Unnik grading system, and the UICC guidelines [18,19]. An overview of clinical and patho-histological data of the leiomyosarcoma and the synovial sarcoma patients is given in Table 1.

**Table 1:** Clinical and immunohistochemical data for leiomyosarcoma and synovial sarcoma patients

Characteristics	Cases	Survivin cytoplasm				Survivin nucleus		
IRS		0-2	3-4	6-8	9-12	0-2	3-4	6-8
Staining		negative	weak	moderate	strong	negative	weak	moderate
**No.**	50	8	15	19	8	29	10	11
**Sex**								
Male	21	4	6	8	3	14	4	3
Female	29	4	9	11	5	15	6	8
**Histological subtype**								
Leiomyosarcoma	24	4	9	10	1	12	8	4
Synovial sarcoma	26	4	6	9	7	17	2	7
**Tumor grade**								
I	5	1	1	2	1	3	1	1
II	21	5	2	10	4	11	6	4
III	24	9	5	7	3	15	3	6
**Tumor stage**								
stage I	5	0	1	3	1	2	2	1
stage II	22	5	3	11	3	15	2	5
stage III	17	2	8	5	2	9	4	4
stage IV	6	1	3	0	2	3	2	1
**Complete resection**								
radical (R0)	38	5	11	15	7	23	7	8
not radical (R1)	12	3	4	4	1	6	3	3
**Localization**								
extremities	33	6	10	10	7	18	7	8
trunc wall	3	0	2	0	1	2	0	1
head/neck	2	1	0	1	0	2	0	0
abdomen/retro-	12	1	3	8	0	7	3	2
peritoneum								
**Patients follow-up**								
alive	18	2	6	7	3	10	6	2
dead	32	6	9	12	5	19	4	9

### Immunohistochemical detection

Immunohistochemical detection was performed as previously described [20,21]. Briefly, the antibody AF886 (RD Systems; Bad Nauheim, Germany, 1:400) was applied to detect survivin protein. Stained specimens were viewed at an objective magnification of ×100 and ×200 by two investigators (HJH and CH). Expression of survivin was determined in the nucleus and in the cytoplasm by assessing semi-quantitatively the percentage of marked tumor cells and the staining intensity. The percentage of positive cells was rated as follows: 1, 1-10% positive cells; 2, 11-50%; 3, 51-80%; and 4, > 80% positive cells. Staining intensity was scored as 1, weak; 2, moderate, and 3, intensive. Scores for percentage of positive cells and scores for expression intensities were multiplied to calculate an immunoreactive score (IRS) [22]; 0-2 = no staining; 3-4 = weak staining; 6-8 = moderate staining; 9-12 = strong staining. We separated the sarcoma patients according to their cytoplasmic expression of survivin in 25% percentile groups: group 1: IRS 0 to 2 (n = 8); group 2: IRS 3 to 4 (n = 15); group 3: IRS 6 to 8 (n = 19); group 4: IRS 9 to 12 (n = 8). To investigate the possibility of an additive effect of cytoplasmic and nuclear expression of survivin on survival we arranged the patients into three groups. In group 1 are all patients (n = 6) whose tumors showed a weak staining of survivin both in the cytoplasm and in the nucleus. In group 2 (n = 35) are all remaining patients; but patients of group 3 (n = 9) with a moderate or strong survivin staining in the cytoplasm and a moderate survivin expression in the nucleus (in both compartments survivin expression showed an immunoreactive score = 6; Table 2). As negative control slides without addition of primary antibody were included for each staining.

**Table 2:** Multivariate Cox's regression hazard analyses

Survivin protein-level	Cytoplasm			Nucleus		
	N	RR	P	N	RR	P
Svv weak (IRS = 3-4)	15	reference		10	reference	
Svv negative(IRS = 0-2)	8	4.2	0.055	29	3.1	0.110
Svv moderate (IRS = 6-8)	19	**5.7**	**0.004**	11	**5.7**	**0.022**
Svv strong (IRS = 9-12)	8	2.4	0.241	none		
						
**Combined Svv protein levels**						
Svv cytopl. & nucleus weak (IRS = 3-4)^1^	6	reference				
Svv all other cases	35	**9.4**	**0.024**			
Svv cytopl. & nucleus moderate+strong (IRS ≥ 6)^2^	9	**24.8**	**0.003**			

Correlation of survivin expression in cytoplasm and/or nucleus with prognosis of leiomyosarcoma and synovial sarcoma patients.

Significant results are in bold face; ^1^Survivin expression both in cytoplasm and in nucleus weak (all IRS = 3-4); ^2^Survivin expression in cytoplasm moderate or strong and survivin expression in nucleus moderate (all IRS ≥ 6)

Abbreviations: Svv-survivin; IRS-immunoreactive score; cytopl.-cytoplasma

### Statistical analyses

Statistical analyses were performed with the SPSS 17.0 software package (SPSS Inc., Chicago, IL). Associations between immunohistochemical stainings and clinical data were calculated with the chi^2^-test. Correlation of expression of survivin with survival was determined in multivariate Cox's regression hazard models (adjusted to tumor stage, tumor localization and type of tumor resection). A p-value of less than 0.05 was considered as statistically significant.

## Results

### Survivin expression in leiomyosarcoma and synovial sarcoma

**Figure 1:**
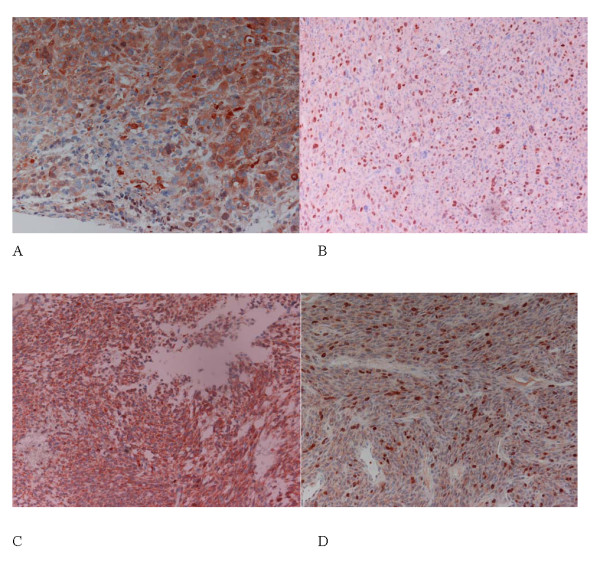
**Immunohistochemical detection of survivin**. Detection of survivin in leiomyosarcoma in cytoplasm (A) and in cytoplasm+nuclei (B) Detection of survivin in synovial sarcoma in cytoplasm (C) and in cytoplasm+nuclei (D) (magnification 200×)

The expression of survivin in the leiomyosarcoma specimens (n = 24) was negative in four, weak in nine, moderate in ten and strong in one cases in the cytoplasm and it was negative in 12, weak in eight, and moderate in four cases in the nucleus, respectively (Table 1; Fig.1). We detected in the synovial sarcoma samples (n = 26) a survivin expression that was negative, weak, moderate or strong in four, six, nine and seven cases in the cytoplasm and that was negative, weak or moderate in 17, two or seven cases, respectively (Table 1; Fig.1). Although, the number of more strongly stained specimens is somewhat higher for synovial sarcomas there was no significant difference in survivin expression between leiomyosarcomas and synovial sarcomas. Therefore, we combined cases of both tumor entities for further statistical analyses. However, future studies in a larger number of leiomyosarcomas and synovial sarcomas have to validate the decision to combine both sarcoma entities in statistical analysis. An association between cytoplasmic and nuclear expression of survivin as trend was found (P = 0.061; chi^2^-test).

### Correlation of survivin expression with clinical data and prognosis

**Figure 2:**
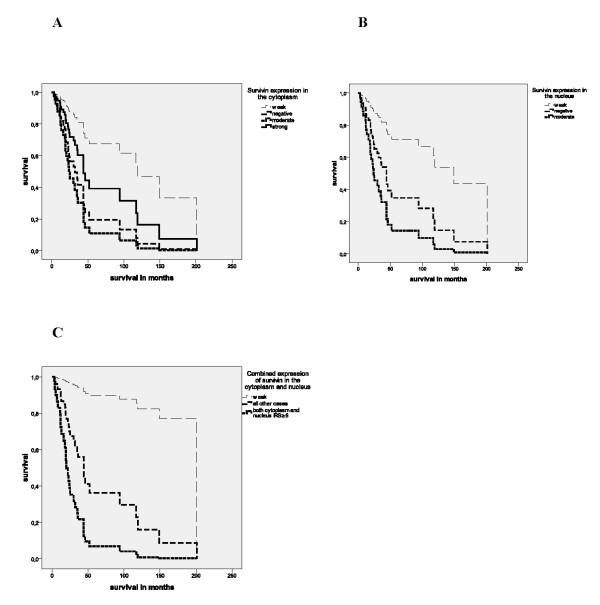
**Multivariate Cox's regression hazard analysis**. Survivin expression in the cytoplasm and/or in nucleus is correlated with a poor prognosis for leiomyosarcoma and synovial sarcoma patients. Survivin expression in the cytoplasm **(A) **and in the nucleus **(B) A**: Upper curve: weak expression; 2^nd ^curve: strong expression; 3^rd ^curve: negative expression and lower curve: moderate expression of survivin in the cytoplasm. **B**: Upper curve: weak expression; 2nd curve: negative expression and lower curve: moderate expression of survivin in the nucleus. Combined expression of survivin in the cytoplasm and in the nucleus **(C) C**: Upper curve: Survivin expression both in cytoplasm and in nucleus weak (all IRS = 3-4); 2^nd ^upper curve: all other cases and lower curve: Survivin expression in cytoplasm moderate or strong and survivin expression in nucleus moderate (all IRS ≥ 6).

Expressions of survivin both in the cytoplasm and in the nucleus were significantly associated with the prognosis of leiomyosarcoma and synovial sarcoma patients in multivariate Cox's regression hazard analyses (adjusted to tumor stage, tumor localization and type of tumor resection). Patients whose tumors expressed survivin in the cytoplasm moderately possessed a 5.7-fold increased risk of tumor-related death (P = 0.004) compared to patients with tumors, that showed a weak expression of survivin (Table 2; Fig. 2A). Expression in the nucleus was again for the patients whose tumors carried a moderate survivin expression associated with a significantly increased risk of tumor-related death (RR = 5.7; P = 0.022; Table 2; Fig. 2B). Next, we studied the possibility of an additive effect of cytoplasmic and nuclear expression of survivin on survival. Patients of group 3 with a moderate or strong survivin staining in the cytoplasm and a moderate survivin expression in the nucleus possessed an additive significantly increased risk of tumor-related death (24.8-fold; P = 0.003; Table 2, Fig. 2C) compared to patients (group1) with a weak expression of survivin both in cytoplasm and nucleus in their tumors. In a Kaplan-Meier analysis patients of group 3 survived on average 29 months whereas patients in group 1 had an average survival time of 73 months but this was not significant because of the limited number of patients in both groups (P = 0.17; data not shown).

## Discussion

Detection of survivin by immunohistochemistry allows distinguishing between survivin expression in the two subcellular pools (cytoplasmic and nuclear). Survivin expression in the cytoplasm could be associated with its control function of cell survival (inhibitor of apoptosis) whereas nuclear staining may rather promote cell proliferation [19]. We investigated both cytoplasmic and nuclear expression of survivin in the soft tissue sarcoma entities leiomyosarcoma and synovial sarcoma. Although, a somewhat higher expression of survivin in synovial sarcomas compared with leiomyosarcomas was detected this difference was not significant. Therefore, we combined tumor samples of both entities for our prognostic evaluations. Both a moderate expression of survivin in the cytoplasm and in the nucleus was correlated with the poorest prognosis of these soft tissue sarcoma patients (RR = 5.7). Remarkably, the patient group with moderate expression of survivin in their tumors includes a higher proportion of retroperitoneal leiomyosarcomas with a poor survival. However, a negative expression of survivin both in the cytoplasm and in the nucleus was, although not significantly, associated with a poor outcome compared with patients whose tumors showed a weak expression of survivin. This finding could be of relevance in planning therapeutic strategies that target survivin. When we combined survivin expression in the cytoplasm and in the nucleus patients whose tumors showed an elevated expression in both compartments carried an additive increased risk of tumor related death (RR = 24.8; P = 0.003) compared to patients with a weak expression of survivin in their tumors. We suggest that survivin expression both in the cytoplasm and in the nucleus should be considered together to evaluate its impact on prognosis.

Recently, the export of nuclear survivin to the cytoplasm could be shown as causal for the survivin-mediated protection against chemo- or radiotherapy-induced apoptosis [23]. Therefore, investigation of survivin expression in different sarcoma entities may have importance for future therapy options. Recently, treatment of rhabdomyosarcoma xenografts with Survivin-shRNA-encoding plasmids showed greater than 70% reduction in growth when compared with control injected tumors [16]. There are several strategies under investigation to target survivin include antisense oligonucleotides, siRNA, ribozymes, immunotherapy and small molecular weight molecules [24]. The translation of these findings to the clinic is currently ongoing with a number of phaseI/II clinical trials including antisense oligonucleotide LY2181308, the low molecular weight molecule inhibitor YM155 and survivin-directed autolougous cytotoxic T lymphocytes [24] The latter strategy, i.e. survivin peptide vaccination w/o different combination therapies has been or is recently applied in different phase I/II clinical trials for advanced melanoma, myeloma, plasma cell neoplasm, pancreatic, colon, cervical, breast, oral cancer and renal cell carcinoma [24-26].

## Conclusion

Altogether inhibition of survivin and other stem cell- associated genes [15] in combination with radio-chemotherapy and/or immunotherapy may help to improve sarcoma therapy in the future.

In summary, both cytoplasmic and nuclear expression of survivin detected by immuno-histochemistry is an independent prognostic factor for leiomyosarcoma and synovial sarcoma patients.

## Abbreviations

STS: soft tissue sarcoma; IRS: immunoreactive score

## Competing interests

All authors disclose any actual or potential conflict of interest including any financial, personal or other relationships with other people or organizations within that could inappropriately influence (bias) our work

## Authors' contributions

HT and K-DS made substantial contributions to conception and design and has been involved in drafting the manuscript. CH and AS, MB, MK, AWE, PW, IM, KH, SH made substantial contributions acquisition of data, and analysis and interpretation of data. H-J H and KH reviewed the pathological diagnosis made substantial contributions to analysis and interpretation of data. All authors read and approved the final manuscript.

## Pre-publication history

The pre-publication history for this paper can be accessed here:

http://www.biomedcentral.com/1471-2407/10/65/prepub
